# Genome-wide association study and subsequent functional analysis reveal regulatory mechanism underlying piglet diarrhea

**DOI:** 10.5713/ab.24.0547

**Published:** 2024-10-28

**Authors:** Dong Chen, Qi Shen, Rui Huang, Zhenjian Zhao, Yang Yu, Shengdi Cui, Junge Wang, Ziyang Chen, Pingxian Wu, Guoqing Tang

**Affiliations:** 1 State Key Laboratory of Swine and Poultry Breeding Industry, College of Animal Science and Technology, Sichuan Agricultural University, Chengdu 625014, China; 2 Key Laboratory of Livestock and Poultry Multi-omics, Ministry of Agriculture and Rural Affairs, College of Animal Science and Technology, Sichuan Agricultural University, Chengdu 625014, China; 3 Farm Animal Genetic Resources Exploration and Innovation Key Laboratory of Sichuan Province, College of Animal Science and Technology, Sichuan Agricultural University, Chengdu 625014, China; 4 Key Laboratory of Agricultural Bioinformatics, Ministry of Education, Sichuan Agricultural University, Chengdu 625014, China; 5 National Center of Technology Innovation for Pigs, Rongchang 402460, China

**Keywords:** Diarrhea, Genome-Wide Association Study (GWAS), Immunity, Inflammatory, Pig

## Abstract

**Objective:**

Piglet diarrhea poses a serious threat to piglet health and the livestock economy, and is one of the most pressing problems in animal husbandry. This study aims to investigate the genetic factors involved in piglet diarrhea and to identify key genes that regulate this condition.

**Methods:**

We screened 600 diarrheal piglets based on unique diarrhea scores for resequencing and conducted a genome-wide association study (GWAS). Through this process, we identified 308 single nucleotide polymorphisms (SNPs) and annotated 151 candidate genes. Extensive functional validation and systematic analysis were performed on key candidate genes *KSR1*, *SKAP1*, *SLC35F6*, and *OR12*.

**Results:**

The study found that the four key genes were involved in the regulation of piglet diarrhea through various mechanisms. *OR12* affects the levels of *ZO-1* and *claudin-1*. Changes in the expression levels of *KSR1* could alter the expression of *IL1-β*, *IL6*, and *TNF-α*, as well as cell migration and proliferation. *SKAP1* could affect the expression of *CD3* and *CD4*, and influence the migration and proliferation ability of cells. *SLC35F6* is involved in cell apoptosis through the *Bcl2/BAX/caspase3* pathway and can also affect mitochondrial membrane potential.

**Conclusion:**

The results of this study provide strong support for breeding programs aimed at disease resistance and offer potential solutions to the problem of piglet diarrhea.

## INTRODUCTION

Diarrhea represents a significant global health challenge, impacting both humans and livestock. Despite extensive research over several decades, diarrhea still affects a large number of organisms each year [1]. Because pigs have metabolic and genetic similarities to humans, they are becoming attractive and accurate biomedical models for disease research [2]. Research on pig diarrhea is expected to enhance our understanding of human diarrhea. Piglets are more susceptible to diarrhea due to their underdeveloped immune and physiological functions, making them an ideal model for studying diarrhea. Piglet diarrhea can be caused by viruses, bacteria, management practices, or climate [3], and is influenced by multiple factors including environment, nutrition, and genetics [4]. Neonatal piglet diarrhea occurs within 3 to 5 days after birth and is the main cause of pre-weaning piglet mortality, while post-weaning diarrhea occurs 3 to 5 days after weaning [5]. Affected piglets exhibit symptoms such as emaciation, dehydration, slow growth, poor development, and vomiting, which can lead to intestinal tissue damage, reduced immunity, decreased nutrient conversion, slow growth, and even a certain proportion of deaths. These reasons make piglet diarrhea one of the most pressing health issues needing resolution.

The process of diarrhea is highly complex and often involves biological regulatory pathways such as inflammatory responses, immune reactions, and apoptosis. Diarrhea can occur from various stimulus signals in the breeding environment, such as food, air, and temperature. During piglet diarrhea, pathogens in the intestine trigger local inflammatory responses which usually serve as a defense mechanism in higher animals against infection and injury. Controlled inflammatory responses are protective reactions of the host, that are capable of eliminating harmful factors and damaged tissue, thereby repairing tissue and restoring normal function [6]. However, excessive inflammatory responses can lead to tissue damage and dysfunction, exacerbating diarrhea [7]. Similarly, immune responses play a crucial role in the process of piglet diarrhea. Immune responses are defense reactions of the body based on interactions among antigens, immune substances, and immune cells against external substances or self-variant substances [8]. During immune responses, pathogen antigens are presented to T cells, which in turn activate T cells and B cells, producing specific antibodies to help eliminate pathogens. When diarrhea occurs, there is active migration and proliferation of new cells and the death of damaged cells in the intestines of piglets. Cell migration and proliferation are core processes in tissue development and disease [9]. Apoptosis is a crucial mechanism for maintaining homeostasis in the body. Appropriate apoptosis helps remove infected or damaged cells, maintaining tissue health. Additionally, intestinal barrier function is closely related to piglet diarrhea [10].

Despite varying degrees of research on the above biological regulatory processes, there is currently no comprehensive regulatory study on piglet diarrhea. The integrated regulation of piglet diarrhea remains a mystery. In this study, a genome-wide association study (GWAS) analysis of diarrhea traits in 600 piglets based on low-depth resequencing data identified 308 significant single nucleotide polymorphisms (SNP) loci significantly associated with piglet diarrhea, and 151 important candidate genes affecting piglet diarrhea were identified. Four key genes related to cellular inflammation and proliferation, cellular immunity, apoptosis, oxidative stress and intestinal barrier were comprehensively screened and identified, including *KSR1*, *SKAP1*, *SLC35F6* and *OR12* genes. Ultimately, a comprehensive analysis of the key regulatory factors affecting piglet diarrhea was conducted based on the results of the study. This study provides a valuable theoretical basis for the analysis of the process of piglet diarrhea.

## MATERIALS AND METHODS

### Ethics approval and consent to participate

The samples for this study were collected from the Xiping Breeding Farm of Jiangyou New Hope Haiboer Breeding Pig Breeding Co. and permission has been obtained from the company.

All experimental procedures conducted in this study adhered to the ethical guidelines and regulations set by the Institutional Review Board (IRB14044) and the Institutional Animal Care and Use Committee of the Sichuan Agricultural University. The study was conducted under the permit number DKY-B20140302, ensuring the appropriate care and use of animals involved in the research.

### Animals

The samples for this study were collected from the Xiping Breeding Farm of Jiangyou New Hope Haiboer Breeding Pig Breeding Co. The piglets were born under confinement conditions between October 1, 2021, and January 1, 2022. During the study period, no pathogenic microorganisms that could cause severe diarrhea in piglets, such as epidemic diarrhea or transmissible gastroenteritis, were detected in the birthing environments. A total of 11,042 piglets were born during the study period, including 8,966 Large White pigs, 1,344 Landrace pigs, and 732 Duroc pigs.

### Phenotype

**Figure 1:**
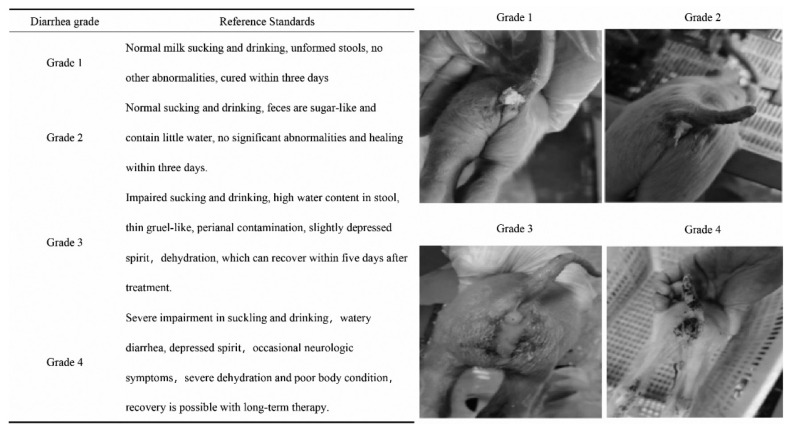
Diarrhea grade and description in piglets.

We organized a professional veterinary team to optimize the diarrhea scoring standards from Ding et al [11] and developed a more comprehensive diarrhea scoring guide (Figure 1). After completing training, a designated member of the veterinary team evaluated and documented the severity of diarrhea in the piglets. A total of 640 piglets developed diarrhea and were scored during the experiment, and 600 samples (453 Large White, 105 Landrace and 42 Duroc) were selected for sequencing. In addition, we collected basic information such as breed, sex, batch, age, weight and room temperature. Finally, three normal piglets and three diarrhea piglets were selected for comparative analysis of intestinal morphology and specific gene expression.

### Sampling

Ear tissue samples were collected from all the 600 piglets and stored in centrifuge tubes containing 75% ethanol. The samples were subsequently stored at −20°C for further analysis. The 6 piglets were euthanized with a 4% pentobarbital sodium solution for comparative analysis, and tissue samples from the duodenum, jejunum, ileum, colon, cecum, and rectum were collected.

### DNA extraction

Ear tissue DNA extraction was performed according to the steps described in the OMEGA Tissue DNA Extraction Kit (D3396-04; Omega Bio-Tek, Norcross, GA, USA) instructions. The extracted DNA samples were assessed for quality using a Nanodrop 2000 Nucleic Acid Protein Analyzer, as well as through 1% agarose gel electrophoresis. For quality assessment, the DNA concentration was measured, and if it exceeded 100 ng/μL and the optical density (OD)206/280 ratio fell within the range of 1.8 to 2.0, indicating a suitable purity level. Additionally, the absence of trailing degradation and the presence of clear bands in the agarose gel electrophoresis further confirmed the good quality of the DNA samples. These qualified DNA samples were then utilized for the subsequent library construction in the sequencing step.

### Genotyping and quality control

A total of 600 individuals, selected from the initial pool of 648 piglets, underwent low-depth resequencing at approximately 1× coverage. The resulting clean data were aligned to the pig reference genome (Sscrofa11.1, Ensembl) using the Burrows-Wheeler Aligner [12] with the parameters mem -t 4 -k 32 -M. To detect SNPs, the Genome Analysis Toolkit [13] was employed, utilizing a Bayesian model. Initially, a total of 10,566,191 SNPs were identified. Subsequently, imputation of the data was performed using a reference population consisting of 735 pigs, and a set of 28,763,360 high-quality SNPs. Quality control for the imputed data was carried out using VCFtools [14] (minor allele frequency [MAF]>0.05, and Hardy-Weinberg equilibrium [HWE] = 10^−5^). Following quality control procedures, a total of 7,545,519 SNP markers passed the criteria and were subsequently utilized for further analysis.

### Genome wide association studies

In this thesis, GWAS analysis was performed for each piglet diarrhea trait using a linear mixed model in GEMMA v0.98.5 software [15]. Prior to the GWAS analysis, a preprocessing step was performed to handle the hierarchical nature of piglet diarrhea. Specifically, the diarrhea phenotype was transformed exponentially using R’s expm1 function. Subsequently, the phenotype was corrected using R’s general linear model (GLM) with a log function as the linkage function. Fixed factors including breed, sex, batch, age, weight, and room temperature were included in the model [16]. The transformation and correction process is outlined as follows:


Y=breed+sex+batch+days+weight+temperature

The obtained correction values were used in the GWAS analysis. The linear mixed model used in the GWAS was as follows:


y=Xm+Wa+e

In the model, ***y*** denotes the corrected phenotype value vector; ***m*** denotes the SNP effect vector; ***a*** denotes the residual polygenic effect vector, obeying the $\left(a\sim MVN\left(0,G\sigma_{a}^{2}\right)\right)$ distribution; ***G*** denotes the genomic relatedness matrix (genomic relatedness matrix [GRM]); ***e*** denotes the residuals, obeying the $\left(a\sim MVN\left(0,G\sigma_{a}^{2}\right)\right)$ distribution, ***I*** denotes the unit vector; ***X*** and ***W*** denote the association matrices of ***m*** and ***a***. GRM according to the following equation:


$$G=\frac{1}{p}\sum_{i=1}^{p}\left(x_{i}-1_{n}{\bar{x}}_{i}\right){\left(x_{i}-1_{n}{\bar{x}}_{i}\right)}^{T}$$

where ***G*** is the kinship matrix between individuals; *n* is the number of individuals; *p* is the number of SNPs; *i* is the *ith* SNP; *X* is the phenotype matrix of *n*×*p*; *x_i_* is the genotype of the *i*th SNP; *χ̄_i_* is the mean of the *i*th SNP. In this analysis, we recommend a significance threshold of 5.5e-6.

To visualize the GWAS results, CMplot v4.0.0, a package available in the R software, was used to generate Manhattan plots. These plots provide a graphical representation of the association results, displaying the genomic positions of SNPs along with their corresponding significance levels.

To assess the presence of false positive signals, the genome inflation factor (λ) was calculated. This factor provides an indication of whether there is an overabundance of significant associations beyond what is expected by chance. In this thesis, the estlambda function from the GenABEL package in R software [17], was utilized to estimate the λ value.

### Candidate gene screening

To identify candidate genes associated with these significant SNPs, we utilized the Ensembl Sscrofa11.1 database (release-112) (www.ensembl.org) and conducted a search within a ±20 kb region surrounding each locus. Initial gene selection was based on GWAS results and functional predictions from National Center for Biotechnology Information (NCBI; https://www.ncbi.nlm.nih.gov/gene/) and GeneCards ( http://www.genecards.org/). We then narrowed down the list of key candidate genes progressively by consulting prior studies and evaluating their expression levels in the intestines of diarrheic and healthy piglets to ensure the precision of functional gene selection.

### Hematoxylin-Eosin staining

Tissue samples approximately 1 cm in length were taken from the duodenum, jejunum, ileum, colon, cecum, and rectum. After being thoroughly rinsed, the tissues were fixed in 10% formalin and then embedded in paraffin. Sections were cut and deparaffinized with xylene, followed by dehydration through a graded series of alcohol. The sections were then stained with hematoxylin and eosin. Finally, the morphological and structural changes of the intestinal sections were observed under a light microscope.

### Cell culture

The Intestinal porcine epithelial cells (IPEC-J2) used in this study was obtained from Cell Line Ontology Subset for Chinese National Infrastructure of Cell Line Resource NICR (Beijing, China). These cells were cultured in a complete medium composed of Dulbecco’s modified eagle medium (DMEM) (Gibco, Grand Island, NY, USA), 10% fetal bovine serum (Gibco) and 1% antibiotics (10 KU/mL, penicillin, 10 mg/mL streptomycin and 25 μg/ mL amphotericin B) (Gibco). The cell culture was maintained in an incubator at 37°C with a continuous supply of 5% CO_2_.

### RNA extraction and real-time quantitative polymerase chain reaction

Take approximately 50 mg of ground intestinal tissue sample or IPEC-J2 cell sample and place it into a 1.5 mL EP tube. Extract RNA using Trizol solution (Invitrogen Corporation, Grand Island, NY, USA) was utilized. The extraction procedure followed the manufacturer’s instructions.

To convert the isolated RNA into complementary DNA (cDNA), we utilized the HiScript III RT SuperMix for quantitative polymerase chain reaction (qPCR) (+gDNA wiper) kit provided by Novozymes. The reverse transcription was conducted following the manufacturer’s recommended protocol.

Subsequently, the cDNA was quantified using the Hieff UNICON Universal Blue qPCR SYBR Green Master Mix from NextGen, which allows for real-time fluorescence measurement. The reaction mixtures were prepared, mixed, and centrifuged according to the instructions provided with the kit. The detection and quantification of the emitted fluorescence signals were then performed using a real-time PCR instrument from BIO-RAD (Hercules, CA, USA).

The primer sequences used for each gene in the quantitative reverse transcription (qRT)-PCR analysis can be found in Table 1.

### Constructing models of inflammatory and immune responses

Lipopolysaccharide (LPS) is a biological macromolecule found in the cell walls of bacteria that can induce an inflammatory response in the body. In animal model studies, LPS is widely used to establish diarrhea models. We treated IPEC-J2 cells with different concentrations of LPS (10 μg/mL, 20 μg/mL) to induce cellular inflammatory responses and construct an inflammatory response model. The supernatant of M1-type macrophages contains substances that participate in the regulation of inflammatory and immune responses. By replacing the culture medium of IPEC-J2 cells with the supernatant of M1-type macrophages, we constructed a cellular immune response model.

### 5-ethynyl-2′-deoxyuridine assay and cell scratch assay

The 5-ethynyl-2′-deoxyuridine (EDU) cell proliferation assay was conducted using the Reebok C10310 kit, following the manufacturer’s instructions. Briefly, the EDU reagent was added to the cell culture medium at a final concentration of 10 μM, and the cells were incubated for 1 hour. The cells were then fixed for 15 minutes and permeabilized for an additional 15 minutes. Subsequently, the cells were incubated with 30 mL of the Click-iT Plus reaction mixture for 5 minutes. The samples were then observed under a fluorescence microscope.

To perform the scratch assay, horizontal lines were drawn on the back of a 6-well plate using a marker pen. At least 5 lines were drawn per well, ensuring they were even and parallel. After 24 hours, a scratch was made using a sterilized 20 μL gun tip or a toothpick, intersecting the marker line on the back of the well plate. The scratched area was observed and imaged under a microscope at 0 and 24 hours.

To quantify the scratch area, Image J software was used to analyze the images and calculate the area of the scratch. This analysis facilitated the evaluation of cell migration or wound healing over time.

### Apoptosis detection and mitochondrial function assay

IPEC-2 cells were dissociated into single cells using trypsin digestion. The cells were then stained with Annexin V-FITC and propidium iodide fluorescent dyes for 10 minutes at room temperature in the dark. Flow cytometry analysis was performed using the CytoFLEX flow cytometer, and the data were analyzed using Kaluza 2.1 software.

The fluorescence intensity of MitoTracker Deep Red serves as an indicator of mitochondrial activity, with stronger fluorescence indicating greater activity. To stain the cells with MitoTracker Deep Red FM, the cells were collected by centrifugation, and the supernatant was carefully removed. The cells were gently resuspended in the pre-warmed MitoTracker Deep Red FM staining working solution and incubated for 15 minutes under normal culture conditions. After the staining was completed, the cells were washed with buffer to remove excess dye. Subsequently, the stained cells were observed under a fluorescent microscope.

### Reactive oxygen species assay

To detect intracellular reactive oxygen species (ROS) using the Dichloro-dihydro-fluorescein diacetate (DCFH-DA) fluorescence probe, the following steps were performed.

First, the cells were collected and the old medium was discarded. Then, the cells were washed twice with pre-warmed phosphate-buffered saline.

Next, a DCFH-DA master solution with a concentration of 10 mM was prepared and diluted to 10 μM using DMEM/F12 medium. Subsequently, 0.2 mL of the diluted DCFH-DA solution was added to each well of a 24-well plate containing the cells.

The plate was placed in an incubator and incubated for 30 minutes. After the incubation period, 1 μL of Hoechst 33342 live cell staining solution (100×) was added to each well and thoroughly mixed. The cells were then incubated for an additional 20 minutes.

Following the incubation, the staining solution was aspirated, and the cells were washed three times with DMEM/F12 medium. Finally, photographs of the stained cells were captured using an inverted fluorescence microscope.

### Immunofluorescence staining

After treatment, the cells were fixed in 4% paraformaldehyde (1 mL) for 1 hour at room temperature. Subsequently, the cells were incubated with 1% bovine serum albumin for 1 hour at room temperature to block nonspecific binding sites. Primary antibodies against *ZO-1* and Claudin (obtained from Servicebio) were then applied, followed by incubation with secondary antibodies, specifically goat anti-rabbit antibodies (obtained from Boster). Additionally, cell nuclei were stained with 4′,6-diamidino-2-phenylindole (obtained from Reebok). Finally, fluorescence signals were visualized and captured using fluorescence microscopy.

### Statistical analysis

The results of the cellular assays were subjected to statistical analysis using GraphPad Prism 8 software. Each test was performed with three replicates, and the data are presented as mean ± standard deviation. The significance level for the statistical tests was set at a p-value of 0.05.

## RESULTS

### Genome-wide association study for piglet diarrhea

**Figure 2:**
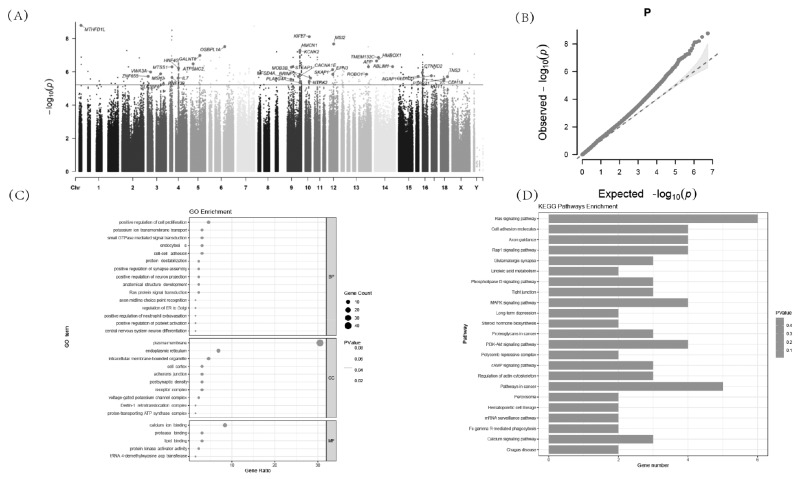
GWAS for piglet diarrhea. (A) The Manhattan plot for the diarrhea piglets in GWAS. (B) The QQ plots of diarrhea piglets’ trait in GWAS. (C) GO enrichment analysis of candidate genes. (D) KEGG enrichment analysis of candidate genes. GO, gene ontology; GWAS, genome-wide association study; KEGG, Kyoto encyclopedia of genes and genomes; QQ, quantile-quantile.

In this study, an association analysis of diarrheal traits was conducted in a total of 600 piglets. The analysis was performed using GWAS based on the SNP data (Figure 2A). As a result, 308 SNPs were found to be significant, and a total of 151 genes were annotated within the ±20 kb genomic region of these loci (Supplementary Table S1). Quantile-Quantile (Q-Q) plot (Figure 2B) suggest that correcting for phenotypes by incorporating fixed effects into a GLM in our dataset reduces the effects of population stratification to some extent. Furthermore, our GWAS results did not deviate significantly from the expected distribution.

To further determine the biological functions of the candidate genes for the identification of diarrhea traits, gene ontology (GO) and Kyoto encyclopedia of genes and genomes (KEGG) analyses (Supplementary Tables S2, S3) were performed on the 151 candidate genes annotated by GWAS in this study (Figures 2C, 2D). GO analysis revealed enrichment in categories such as plasma membrane, positive regulation of cell proliferation, endoplasmic reticulum, calcium ion binding, and signal transduction. KEGG enrichment analysis showed enrichment in pathways including Ras signaling pathway, Pathways in cancer, Calcium signaling pathway and mitogen-activated protein kinase (MAPK) signaling pathway. These results shed light on the potential functional roles of the candidate genes in the context of diarrheal traits in piglets.

To further resolve the process of piglet diarrhea, we screened 38 genes that may be associated with piglet diarrhea based on the results of functional annotation of candidate genes (Table 2). Four important candidate genes including *KSR1*, *SKAP1*, *SLC35F6* and *OR12* were obtained by screening based on various literatures and previous research experience.

### Intestinal changes in piglets with diarrhea

**Figure 3:**
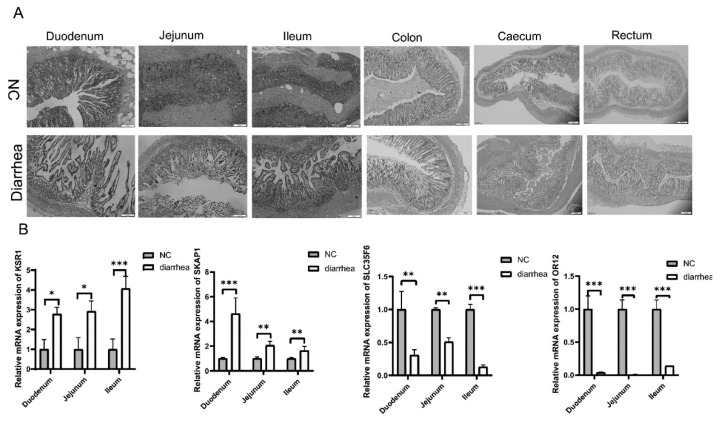
Intestinal changes in piglets with diarrhea. (A) Hematoxylin and eosin (H&E) were demonstrated in six intestinal analyses of diarrhea piglets and normal piglets, including duodenum⊠ jejunum, ileum, colon, caecum, rectum. (B) *KSR1, SKAP1, SLC35F6, OR12* mRNA expression in diarrhea piglets and normal piglets. NC, negative control. * p<0.05, ** p<0.01, *** p<0.001.

Histological sections stained with hematoxylin and eosin staining showed noticeable morphological differences in the duodenum, jejunum, ileum, colon, cecum, and rectum between diarrheic and normal piglets (Figure 3A).

Compared to normal piglets, diarrheic piglets exhibited varying degrees of damage to intestinal tissue integrity, including misaligned villi, blunted villi, and reduced crypt depth. These observations suggest compromised intestinal health in diarrheic piglets compared to their normal counterparts.

Furthermore, we conducted qRT-PCR analysis to assess the expression levels of four genes (*KSR1*, *SKAP1*, *SLC35F6* and *OR12*) in the small intestine of both diarrheic and normal piglets (Figure 3B). Our research results indicate that the expression levels of the *KSR1* and *SKAP1* genes are significantly upregulated, while the expression levels of the *SLC35F6* and *OR12* genes are significantly downregulated in piglets with diarrhea. This suggests their potential involvement in the pathological processes associated with diarrhea.

These findings highlight the association between altered gene expression and intestinal dysfunction in diarrheic piglets, shedding light on potential molecular mechanisms underlying the development of diarrhea in pigs.

### Effects of *KSR1* knockdown and overexpression on inflammatory and proliferation in IPEC-J2 cells

**Figure 4:**
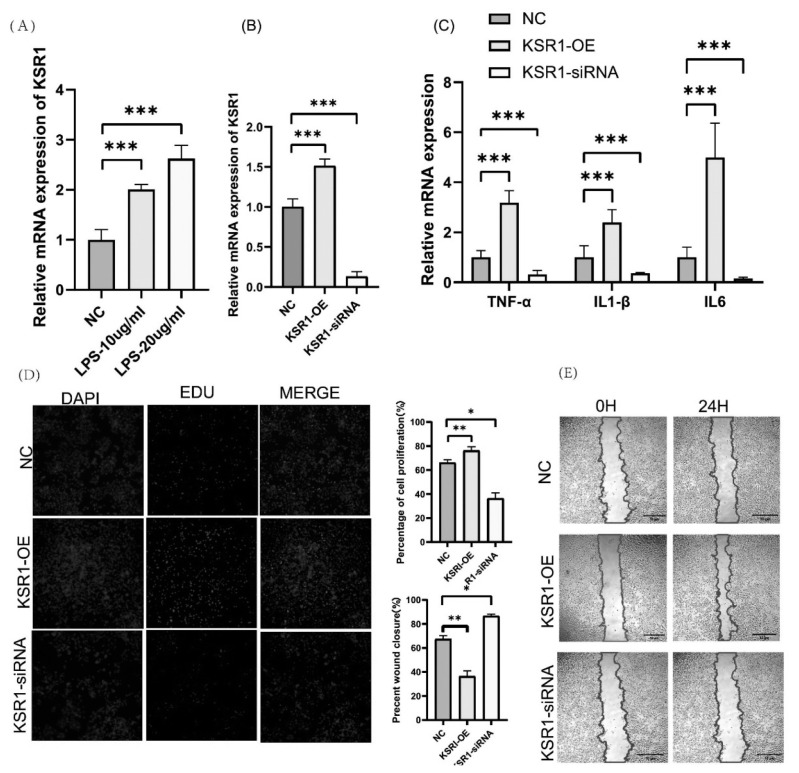
Effects of knockdown and overexpression of *KSR1* on inflammatory, proliferation and apoptosis in IPEC-J2 cells. (A) The mRNA expression of *KSR1* after LPS (10 μg/mL, 20 μg/mL) treatment of IPEC-J2. (B) The mRNA expression of *KSR1*-OEand *KSR1*-siRNA. (C) The mRNA expression of *TNF*-α, *IL1-β* and *IL6* in IPEC-J2 cells by *KSR1* overexpression and knockdown. (D) EdU results of KSR1-OE, KSR1-siRNA and NC at 24h in IPEC-J2 cells. EdU marks the proliferating cells, Hoechest represents the nucleus, and Merge represents an overlay of EdU and Hoechest. (E) Scratch assays and quantitative analysis were performed to detect the migration of IPEC-J2 cells after treatment with *KSR1* knockdown and overexpression for 24 h. NC, negative control; LPS, lipopolysaccharide; IPEC, intestinal porcine epithelial cells; EdU, 5-ethynyl-2′-deoxyuridine; TNF, tumor necrosis factor; IL, interleukin. * p<0.05, ** p<0.01, *** p<0.001.

We studied *KSR1*’s impact on IPEC-J2 cell inflammation. After LPS exposure, *KSR1* gene expression in IPEC-J2 cells rose markedly above control levels (Figure 4A), indicating its potential in modulating inflammation. To explore this further, we performed *KSR1* gene overexpression and knockdown studies (Figure 4B). The overexpression of *KSR1* resulted in a significant upregulation of pro-inflammatory genes, such as *IL1-β*, *IL6*, and *TNF-α*, while the knockdown of *KSR1* led to a significant decrease in their expression levels (Figure 4C). These findings indicate that *KSR1* may contribute to the promotion of inflammation in IPEC-J2 cells.

Additionally, we investigated the impact of *KSR1* on cell proliferation and apoptosis in IPEC-J2 cells. The EDU cell proliferation assays revealed that knockdown of *KSR1* inhibited cell proliferation, whereas overexpression of *KSR1* promoted cell proliferation (Figure 4D). Furthermore, the cell migration rate did not show a significant difference after *KSR1* knockdown compared to the negative control (NC), but it was significantly increased following *KSR1* overexpression (Figure 4E). These results provide insights into the dual roles of *KSR1* in modulating inflammation and cell proliferation in IPEC-J2 cells. The findings suggest that *KSR1* may act as a regulator of inflammatory responses and cellular proliferation, highlighting its potential importance in maintaining gastrointestinal health.

### Effects of *SKAP1* knockdown and overexpression on immunization and proliferation in IPEC-J2 cells

**Figure 5:**
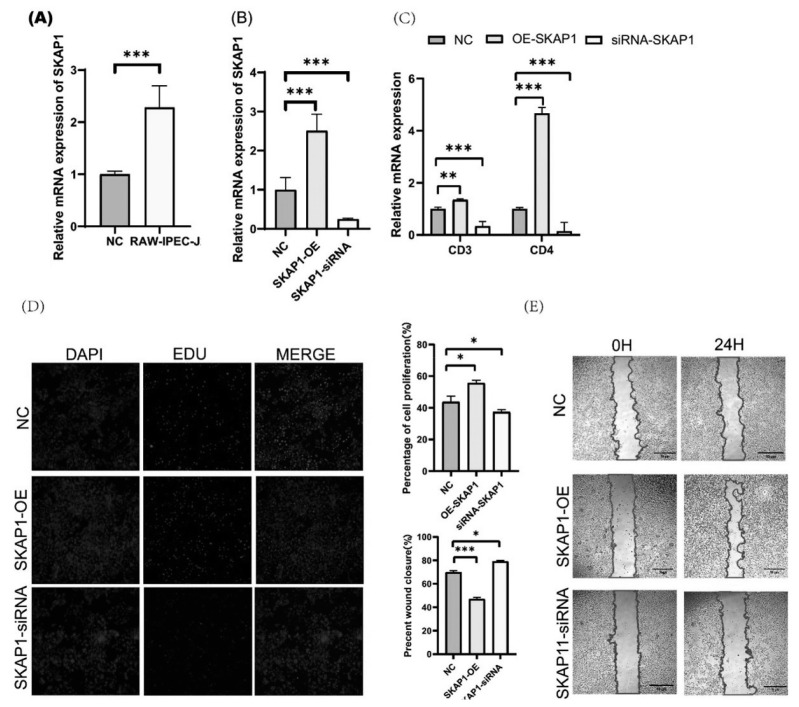
Effects of knockdown and overexpression of *SKAP1* on immunization, proliferation and apoptosis in IPEC-J2 cells. (A) The mRNA expression of *KSR1* after M1 macrophage supernatant for 24 hours in IPEC-J2 cells. (B) The mRNA expression of *SKAP1*-OE and *SKAP1*-siRNA. (C) The mRNA expression of *SKAP1*-OE and *SKAP1*-siRNA. (C) The mRNA expression of *CD3* and *CD4* in IPEC-J2 cells by *SKAP1* overexpression and knockdown. (D) EdU results of *SKAP1*-OE, *SKAP1*-siRNA and NC at 24h in IPEC-J2 cells. EdU marks the proliferating cells, Hoechest represents the nucleus, and Merge represents an overlay of EdU and Hoechest. (E) Scratch assays and quantitative analysis were performed to detect the migration of IPEC-J2 cells after treatment with *SKAP1* knockdown and overexpression for 24 h. NC, negative control; DAPI, 4′,6-diamidino-2-phenylindole; EdU, 5-ethynyl-2′-deoxyuridine; IPEC, intestinal porcine epithelial cells. * p<0.05, ** p<0.01, *** p<0.001.

After treatment with M1 macrophage supernatant for 24 hours, we observed a significant increase in *SKAP1* gene expression (Figure 5A). This suggests that *SKAP1* may be involved in immune-related processes in IPEC-J2 cells. To further explore the role of *SKAP1* in immune responses, we conducted overexpression and knockdown experiments of the *SKAP1* gene (Figure 5B). The overexpression of *SKAP1* resulted in a significant increase in *CD3* and *CD4* expression levels, indicating its potential role in modulating immune-related gene expression (Figure 5C). Conversely, the knockdown of *SKAP1* led to a significant decrease in *CD3* and *CD4* expression levels, further highlighting its involvement in immune regulation.

Additionally, we examined the impact of *SKAP1* on cell proliferation using the EDU cell proliferation assay. The results showed that knockdown of *SKAP1* inhibited cell proliferation, while overexpression of *SKAP1* promoted cell proliferation (Figure 5D). Moreover, our findings demonstrated that *SKAP1* overexpression significantly increased cell migration velocity, whereas *SKAP1* knockdown did not result in a significant difference in cell migration velocity compared to the control group (Figure 5E).

These findings emphasize the potential role of *SKAP1* in modulating immune-related gene expression and cellular processes, such as proliferation and migration, in IPEC-J2 cells. The results suggest that *SKAP1* may be involved in regulating immune responses and cell behavior, highlighting its potential importance in maintaining gastrointestinal health.

### Effects of *SLC35F6* knockdown and overexpression on apoptosis in IPEC-J2 cells

**Figure 6:**
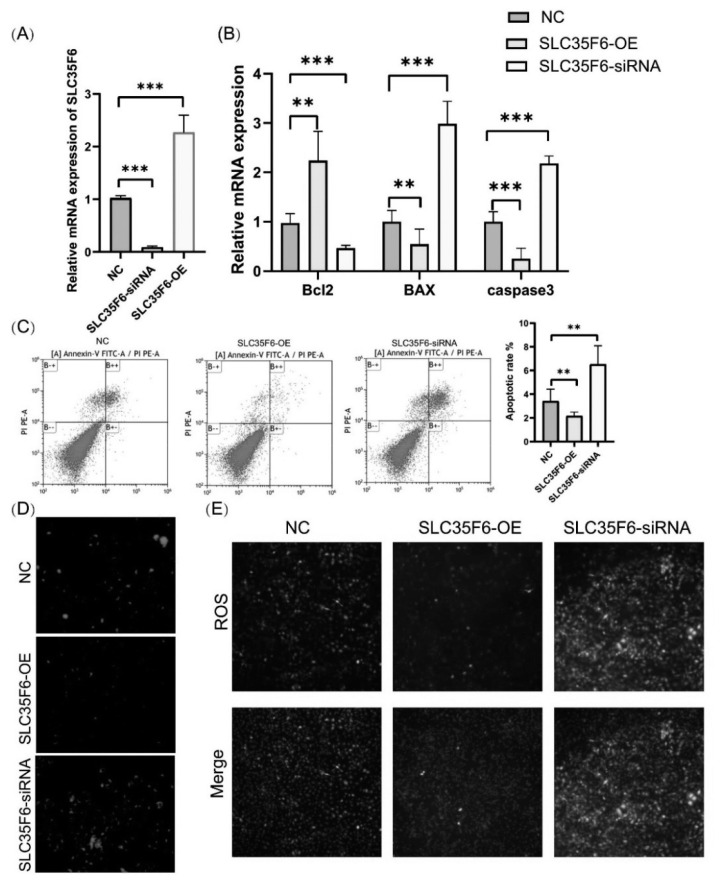
Effects of knockdown and overexpression *SLC35F6* on apoptosis in IPEC-J2 cells. (A) The mRNA expression of *SLC35F6*-OE and *SLC35F6*-siRNA. (B) The mRNA expression of *Bcl2*, *BAX* and *caspase3* in IPEC-J2 cells by *SLC35F6* overexpression and knockdown. (C) Apoptosis was verified through flow cytometry with *SLC35F6*-OE and *SLC35F6*-siRNA treatment. (D) MitoTracker Red with *SLC35F6*-OE and *SLC35F6*-siRNA treatments. The ROS level was measured using MitoTracker Red, a reduced mitochondrial dye that does not fluoresce until it is oxidized by ROS. (E) Detection of *SLC35F6* knockdown or overexpression of ROS using the fluorescent probe DCFH-DA. NC, negative control; ROS, reactive oxygen species; IPEC, intestinal porcine epithelial cells; DCFH-DA, dichloro-dihydro-fluorescein diacetate. ** p<0.01, *** p<0.001.

In our study, we aimed to investigate the impact of *SLC35F6* on apoptosis in IPEC-J2 cells. To further understand the role of *SLC35F6* gene, we conducted overexpression and knockdown experiments of the *SLC35F6* gene (Figure 6A). Through the overexpression of the *SLC35F6* gene, we observed a significant increase in the expression levels of the apoptosis-inhibiting *Bcl2* gene, while the expression levels of *BAX* and *caspase3*, associated with apoptosis, were significantly decreased (Figure 6B). These findings suggest that *SLC35F6* may have an anti-apoptotic effect in IPEC-J2 cells.

To further evaluate the effect of *SLC35F6* on apoptosis, we utilized the Membrane Linker V/FITC Apoptosis Kit. Our results showed that knockdown of the *SLC35F6* gene led to a significant increase in the percentage of total apoptosis compared to the control group. Conversely, overexpression of the *SLC35F6* gene resulted in a significant reduction in the total percentage of apoptosis in IPEC-J2 cells (Figure 6C).

These findings suggest that *SLC35F6* may play a role in regulating apoptosis in IPEC-J2 cells, with its overexpression potentially promoting cell survival by upregulating the apoptosis-inhibiting gene *Bcl2* and downregulating pro-apoptotic genes such as *BAX* and *caspase3*. Further investigations into the molecular mechanisms underlying the anti-apoptotic effects of *SLC35F6* could provide valuable insights into its potential as a therapeutic target in the context of intestinal health.

Additionally, we investigated the impact of *SLC35F6* gene overexpression on mitochondrial membrane potential in cells. Figure 6D presents the experimental results of assessing mitochondrial membrane potential using the MitoTracker fluorescence probe. It is evident that upon *SLC35F6* gene knockdown, the fluorescence intensity increased, signifying a significant enhancement in mitochondrial membrane potential when compared to the control group. Conversely, following the overexpression of the *SLC35F6* gene, the fluorescence intensity decreased, indicating a reduction in mitochondrial membrane potential.

To measure the level of ROS in IPEC-J2 cells, we used an ROS assay reagent. The results revealed that the fluorescence intensity of ROS was notably greater in the *SLC35F6* knockdown group than in the control group. On the other hand, the fluorescence intensity of ROS was significantly lower in the *SLC35F6*-overexpressing group than in the control group (Figure 6E).

### Effects of *OR12* knockdown and overexpression on tight junctions in IPEC-J2 cells

**Figure 7:**
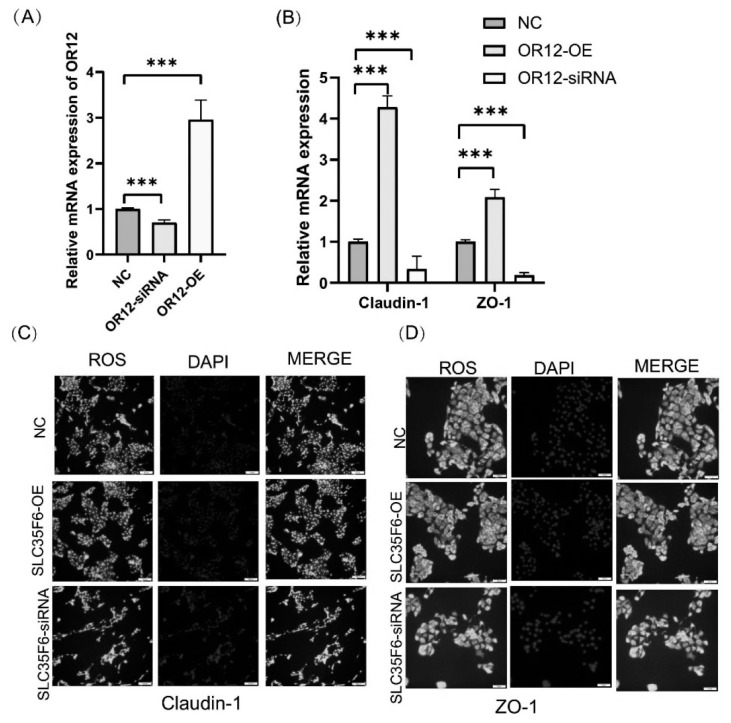
Effects of knockdown and overexpression *OR12* on tight junctions in IPEC-J2 cells. (A) The mRNA expression of *SLC35F6*-OE and *SLC35F6*-siRNA. (B) The mRNA expression of *Claudin-1* and *ZO-1* in IPEC-J2 cells by *SLC35F6* overexpression and knockdown. (C) Differential *Claudin-1* protein expression after *KSR1* overexpression and knockdown in IPEC-J2 cells (100×). (D) Differential *ZO-1* protein expression after *KSR1* overexpression and knockdown in IPEC-J2 cells (200×). C, negative control; ROS, reactive oxygen species; DAPI, 4′,6-diamidino-2-phenylindole; IPEC, intestinal porcine epithelial cells. *** p<0.001.

In order to uncover the role of *OR12* in the intestine, we performed overexpression and knockdown of *OR12* and conducted real-time qPCR experiments. The qPCR results revealed a decrease in the expression of *ZO-1* and *Claudin-1* genes following the knockdown of *OR12* genes. Conversely, the overexpression of *OR12* led to an increase in the expression of *ZO-1* and *Claudin-1* (Figure 7B).

Furthermore, immunofluorescence experiments were conducted to complement the quantitative findings. Consistent with the qPCR results, the immunofluorescence results demonstrated disrupted tight junctions (TJs) in IPEC-J2 cells following *OR12* knockdown. Notably, the expression of *Claudin-1* and *ZO-1* were significantly reduced. Conversely, when *OR12* were overexpressed, the impact on TJs was not as pronounced compared to the control group, although the difference was not statistically significant (Figures 7C, 7D).

Overall, these findings suggest that the modulation of *OR12* can influence the expression of TJ proteins, thereby impacting the integrity of TJs in IPEC-J2 cells. This highlights the potential role of *OR12* in regulating the function of TJs and maintaining intestinal barrier integrity.

## DISCUSSION

In our study, we introduced a novel diarrhea scoring scale designed to characterize diarrhea traits in GWAS. This is the first scoring system to integrate piglet fecal characteristics with piglet health status. For genomic data collection, we initially opted for low-depth whole-genome resequencing with 1× coverage. However, the SNP calling results revealed continuous gaps in the SNP regions, highlighting the limitations of using 1× coverage for low-depth resequencing in GWAS analysis. Despite applying rigorous quality control and imputation (DR^2^ = 0.91), this may still have affected our GWAS findings to some extent. To minimize potential false-positive signals in the GWAS results, we lowered the significance threshold and identified candidate genes based on literature evidence and prior research experience. Using this approach, we identified 308 significant loci, and among the 151 diarrhea-related genes, we selected OR12, *KSR1*, *SKAP1*, and *SLC35F6* for functional validation. Initial comparisons showed significant differences in the expression of these genes between diarrheal and normal piglets’ intestines, suggesting their potential role in piglet diarrhea. Subsequently, we conducted functional validation experiments and found that these four genes are involved in the regulation of cell barriers, inflammatory responses, immune responses, and apoptosis in IPEC-J2 cells. This is the first report demonstrating the integrated regulation of OR12, *KSR1*, *SKAP1*, and *SLC35F6* in piglet diarrhea.

*OR12* belongs to the olfactory receptor family. However, the expression of receptors encoded by the OR gene family is not limited to the olfactory epithelium but is also found in non-olfactory organs such as the intestine [18]. Studies have shown that odorants in the intestinal lumen may influence pathological conditions like diarrhea and irritable bowel syndrome through their interaction with olfactory receptors [19]. Consequently, we conducted downstream functional validation of *OR12* to investigate its potential role. The results from real-time quantitative fluorescence experiments and supporting evidence from immunofluorescence experiments indicated that knockdown of the *OR12* gene significantly reduced the expression of *Claudin-1* and *ZO-1* in IPEC-J2 cells. Reports have shown that the expression of *ZO-1* and *Claudin-1* is significantly reduced in the intestines of diarrhea patients [20], which supports our findings. ZO proteins are scaffold proteins that play a critical role in the formation of TJs. Membrane-associated ZO proteins can promote the recruitment and enrichment of TJ proteins such as *Claudin-1*, cytoskeletal junction proteins, and regulatory factors, leading to the formation of TJ complexes [21]. Studies have shown that the high-efficiency barrier function of intestinal epithelial cells is provided by TJ proteins (especially the claudin family), and changes in the abundance or molecular structure of TJ proteins can alter cell permeability, disrupt barrier function, and increase the risk of diarrhea [22].

Piglet diarrhea is often accompanied by an inflammatory response. Controlled inflammatory responses help the body resist pathogens, but excessive inflammation can damage intestinal tissue. *KSR1* as a cascade scaffold of extracellular regulated protein kinases (ERK), can participate in the regulatory interactions of Raf and ERK by enhancing and weakening ERK cascade activation [13], affecting MAPK signaling [23], and thus participating in the body’s inflammatory response [24]. The results showed that the expression of *KSR1* was significantly increased in LPS-induced IPEC-J2 cells. *KSR1* overexpression led to a significant increase in the levels of pro-inflammatory factors *IL-1β*, *IL-6*, and *TNF-α*. This indicates that upregulation of the *KSR1* gene promotes the expression of *IL-1β*, *IL-6*, and *TNF-α*. There are few reports on the direct relationship between these three inflammatory factors and the MAPK pathway, and they seem to be more closely related to the nuclear factor kappa-B (NF-κB) pathway [25–27]. Similar results were obtained by Docena et al [28], but in their study, MAPK inhibition led to similar suppression levels of *IL-1β* and *IL-6* expression. Our study showed that the changes in *IL-6* were more significant than those in *IL-1β*. NF-κB is a key transcription factor in the immune response [29]. NF-κB in cells is isolated outside the nucleus by IκB and can be activated by *TNF-α* and *IL-1β* [30]. Activated NF-κB can induce the expression of inflammatory genes, including *TNF-α*, *IL-1β*, *IL-6*, *IL-12p40*, and cyclooxygenase-2 [31,32], promoting the production of these inflammatory cytokines. Additionally, Meng et al’s study [33] showed that *IL-1β* induces an increase in *IL-6* within NF-κB. Therefore, we speculate that during diarrhea, upregulation of the *KSR1* gene activates the MAPK signaling pathway, leading to the production of *TNF-α* and *IL-1β* inflammatory factors, which may participate in the activation of the NF-κB signaling pathway, enhancing the transcription and expression of *TNF-α*, *IL-1β*, and *IL-6* inflammatory factors. This positive feedback loop induces and rapidly amplifies inflammation. Controlled inflammation occurs in some areas, effectively combating infection or injury and repairing itself. When the inflammatory response cannot stop, excessive inflammation damages the body, even exacerbating diarrhea.

Immune response is the body’s defensive reaction against diarrhea, and we found that one of the candidate genes in the regulation of piglet diarrhea, *SKAP1*, is an immune connector and notably, *SKAP1* exhibited the highest expression in all tissues within the small intestine. When we cultured IPEC-J2 cells with macrophage supernatant after macrophage M1 polarization, we found that *SKAP1* expression was upregulated. *SKAP1* is known as a major binding partner of Adhesion and degranulation-promoting adapter protein (ADAP) in T cells [34] and is involved in immune cell junctions [35]. Furthermore, our research revealed a significant increase in the expression levels of *CD3* and *CD4* following *SKAP1* overexpression. *CD3* is a marker for mature total T lymphocytes, while *CD4* is associated with the regulation of the immune response of helper T cells [36]. Both *CD3* and *CD4* hold crucial roles in the immune response and exhibit multifaceted functions in defense against various threats [37]. Therefore, we speculate that *SKAP1* may regulate the immune response by affecting the expression of *CD3* and *CD4*, thereby influencing T cell antigen recognition.

Additionally, through EDU cell proliferation experiments on *KSR1* and *SKAP1*, we found that Knockdown *SKAP1* and *KSR1* genes inhibited cell proliferation compared to the NC group, while overexpressing *SKAP1* and *KSR1* genes promoted cell proliferation. This may be because the proliferation of intestinal epithelial cells plays an important role in maintaining the integrity of the intestinal barrier, and the damage caused by diarrhea stimulates the proliferation of intestinal epithelial cells to repair the damaged barrier [38].

Finally, apoptosis is a pervasive mode of cell death used to remove unwanted, injured, or virologically infected cells. There is little research on *SLC35F6*, and it has been reported that the protein encoded by *SLC35F6* is involved in maintaining mitochondrial membrane potential and apoptosis [39]. In our study, we found that *SLC35F6* expression is downregulated in the gut of diarrheal piglets. We investigated the effect of *SLC35F6* on apoptosis and demonstrated its critical regulatory role. Apoptosis is regulated by a balance between anti-apoptotic and pro-apoptotic factors, with the cysteine aspartate-specific protease (Caspase) family playing a central role [40]. The complex interaction network between pro-survival (*BCL-2*, *BCL-XL*, and *MCL-1*) and pro-death (*BIM*, *BAD*, *BAK*, and *BAX*) *BCL-2* family proteins can control programmed cell death, and downregulating the *Bcl-2/BAX* ratio can increase caspase-3 expression [41]. In our study, after overexpressing and Knockdown the *SLC35F6* gene, we found that the *Bcl2/BAX* ratio and *Caspase3* expression significantly decreased and increased, respectively, indicating that knocking down the *SLC35F6* gene promotes apoptosis. Additionally, our study showed that overexpressing the *SLC35F6* gene significantly weakened mitochondrial membrane potential and reduced ROS levels in cells. Knockdown of the *SLC35F6* gene significantly enhanced mitochondrial membrane potential and increased ROS levels in cells. ROS in cells can mediate certain apoptotic responses, and excessive ROS levels can also directly damage DNA to promote apoptosis [42].

**Figure 8:**
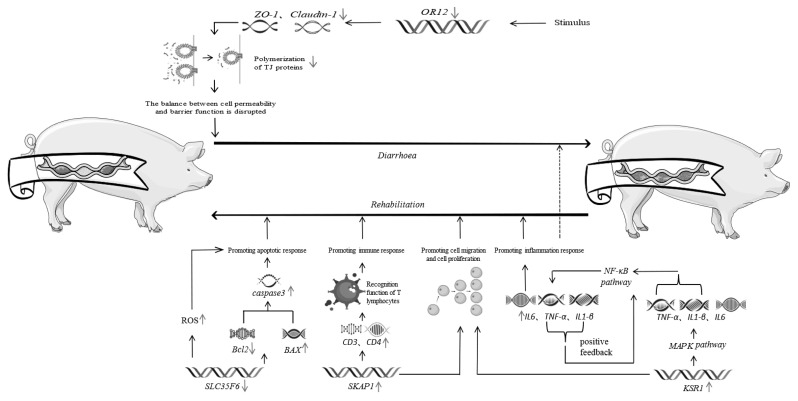
Integrated regulation of piglet diarrhea.

In summary, through the screening of candidate genes obtained by GWAS and functional validation of these genes, we preliminarily studied and analyzed the integrated regulation of piglet diarrhea by the four genes OR12, *KSR1*, *SKAP1*, and *SLC35F6* (Figure 8). The downregulation of *OR12* due to certain environmental or organismal stimulus signals, which in turn leads to the downregulation of *ZO-1* and *Claudin-1*, disrupts the relationship between paracellular permeability and barrier function and increases the risk of diarrhea occurrence. When the intestine is damaged and invaded by pathogens. the *KSR1* gene is upregulated and the MAPK signaling pathway is activated, producing inflammatory factors such as *TNF-α*, *IL-1β* and *IL-6*, where *TNF-α* and *IL-1β* in turn activate the NF-κB signaling pathway, enhancing the transcriptional expression of inflammatory factors such as *TNF-α*, *IL-1β* and *IL-6*. Inflammation is induced and rapidly amplified through a positive feedback loop. A controlled inflammatory response occurs in some areas, which in turn effectively fights infection or damage and repairs itself. When the inflammatory response cannot be stopped, the excessive inflammatory response can damage the organism and even exacerbate the diarrhea condition. At the same time, *SKAP1* gene affects the expression of *CD3* and *CD4*, which in turn affects the antigen recognition function of T-cells and thus participates in the regulation of the immune response against diarrhea. In addition, *SLC35F6* gene participates in the *Bcl-2/BAX/Caspase-3* signaling pathway, which is down-regulated, leading to a decrease in the ratio of *BCL-2* and *BAX*, and promotes the activation of *caspase-3*, thereby promoting apoptosis of damaged cells and the recovery of the organism. It is important to emphasize that the process of diarrhea involves multiple factors and is highly complex. The aforementioned findings are based on our GWAS and subsequent functional validation studies. We will continue to further verify and explore these results.

## CONCLUSION

Through GWAS and post-GWAS functional validation and analyses, this study demonstrated for the first time that *OR12* is an important gene for protecting intestinal barrier function. In addition, we found that *KSR1*, *SKAP1*, and *SLC35F6* can participate in the regulation of piglet diarrhea through inflammatory response, immune response, cell proliferation and migration, and apoptosis. These results not only extend our understanding of the diarrheal process, but also provide evidence for comprehensive regulatory studies of piglet diarrhea.

## Figures and Tables

**Table 1:** Sequences and parameters of the primers used for qRT-PCR

Gene (abbreviation)	Sequence (5′ → 3′)
*β-actin*	F:AAGGACCTCTACGCCAACAC R:CTGGCTGATCCACATCTGCT
*KSR1*	F:CATTTGAGCTGGAGCACACC R:TGCGGTGAAGCTCTGTACTT
*SKAP1*	F:TGTTGGCTCCTGGAAGATGC R:TTTGCTGAAAGCCCCGTAGA
*SLC35F6*	F:ATAGACAGCCATGTGGCGAG R:AAAGAAGGGGGTTGAAGGGC
*OR12*	F:CTTCTCCCTGCTGGCAACTTC R:GGCACATGGTGATGGGATAGT
*IL-1β*	F:CGAACCCGTGTTGCTGAAGGAG R:TGGATGGGCGGCTGATTTGAAG
*IL-6*	F:TGGTGGCTTTGTCTGGATTC R:ATCAGGAGACCTGCTTGATG
*TNF-α*	F:GGCCCAAGGACTCAGATCAT R:GGCATACCCACTCTGCCATT
*CD-3*	F:TCATCGCATGGCTACTCACC R:ACCTCTGCCAGCATTTACCC
*CD-4*	F:ACCTTCTGGAAGCTTGCCTCT R:TTTCCTGTCAGCTCAGGGAGG
*BCL-2*	F:GACTTTGCCGAGATGTCCAG R:ACGCTCTCCACACACATGAC
*BAX*	F:TGCATGGTGCCCTCTTGATT R:TGCCGTCAGCAAACATTTCG
*Caspase-3*	F:GGATTGAGACGGACAGTGGG R:CCGTCCTTTGAATTTCGCCA
*ZO-1*	F:ACCCACCAAACCCACCAA R:CCATCTCTTGCTGCCAAACTATC
*Claudin-1*	F:TGATGAGGTGCAGAAGATGC R:CCAGTGAAGAGAGCCTGACC

qRT-PCR, quantitative reverse transcription polymerase chain reaction.

**Table 2:** List of important candidate genes related to Diarrhea

Ensembl gene ID	Hgnc symbol	Chromosome	Start position	End position	Function
ENSSSCG00000004092	*MTHFD1L*	1	15140929	15330265	Apoptosis
ENSSSCG00000008428	*MSH2*	3	93082958	93163556	inflammation
ENSSSCG00000008562	*SLC35F6*	3	112280654	112296874	Apoptosis
ENSSSCG00000025266	*VWA3A*	3	24028836	24097582	Blood clotting
ENSSSCG00000007617	*ZNF655*	3	6504699	6521051	Apoptosis
ENSSSCG00000034242	*HNF4G*	4	60505388	60641052	Tight junctions
ENSSSCG00000006161	*IL7*	4	57677895	57744713	inflammation
ENSSSCG00000005975	*MTSS1*	4	14933290	15103256	Cell Proliferation
ENSSSCG00000024412	*RNF139*	4	15157571	15182520	Cell Proliferation
ENSSSCG00000062561	*ATP5MC2*	5	18871025	18879933	Respiratory electron transfer
ENSSSCG00000000718	*GALNT8*	5	65697120	65735521	Intestinal Barrier
ENSSSCG00000003712	*OSBPL1A*	6	108997730	109241950	Metabolism
ENSSSCG00000015515	*BRINP2*	9	118967670	119095345	Cell Proliferation
ENSSSCG00000015543	*CACNA1E*	9	122827386	123318372	immunity
ENSSSCG00000035595	*HMCN1*	9	126850208	127342696	immunity
ENSSSCG00000023451	*KCNK2*	9	128429232	128659662	Signaling
ENSSSCG00000021487	*MFSD4A*	9	66249434	66292482	Material transport
ENSSSCG00000023351	*PLA2G4A*	9	127853581	128164825	Cell proliferation, inflammation
ENSSSCG00000015301	*STEAP1*	9	8996943	8997498	Apoptosis
ENSSSCG00000021571	*KIF27*	10	31026133	31117900	Material transport
ENSSSCG00000038811	*MOB3B*	10	38437933	38694667	Apoptosis
ENSSSCG00000010959	*NTRK2*	10	30030050	30429938	Apoptosis
ENSSSCG00000027226	*EPN3*	12	26812653	26825485	Cell Proliferation
ENSSSCG00000038505	*MSI2*	12	33537163	33974845	Cell cycle regulation
ENSSSCG00000017527	*SKAP1*	12	24371699	24680280	immunity
ENSSSCG00000017753	*KSR1*	12	43927222	44135833	inflammation
ENSSSCG00000012022	*APP*	13	189434854	189716120	Cell migration
ENSSSCG00000012001	*ROBO1*	13	175348223	176479482	Cell migration
ENSSSCG00000010651	*ABLIM1*	14	124759811	125108722	Immunity, inflammation
ENSSSCG00000009682	*HMBOX1*	14	12692607	12895078	Immunity, inflammation
ENSSSCG00000009753	*TMEM132C*	14	26209553	26611549	Material transport
ENSSSCG00000016317	*AGAP1*	15	135442440	135999162	Material transport
ENSSSCG00000026842	*CDH18*	16	8090253	9117046	Cell proliferation, inflammation
ENSSSCG00000016780	*CTNND2*	16	508630	1521773	Cell Proliferation
ENSSSCG00000006966	*CLDN23*	17	483635	484686	Tight junctions
ENSSSCG00000016597	*POT1*	18	22885704	22981893	immunity
ENSSSCG00000016614	*PTPRZ1*	18	25046027	25228276	immunity
ENSSSCG00000016725	*TNS3*	18	48960938	49192434	Cell Proliferation

## SUPPLEMENTARY MATERIAL

**Supplementary Table S1.** Top SNPs associated with diarrhea in pig

**Supplementary Table S2.** GO enrichment analysis of candidate genes (MF)

**Supplementary Table S3.** KEGG enrichment analysis of candidate genes
