# Supplementary material for: Genome-wide association study and subsequent functional analysis reveal regulatory mechanism underlying piglet diarrhea
Source: Anim Biosci. 2024 Oct 28;38(4):612–28. doi: 10.5713/ab.24.0547 (PMC11917426; doi:10.5713/ab.24.0547)
Supplement: Supplementary file 1 [file ab-24-0547-Supplementary-Table-1.pdf]

Supplementary table S1. Top SNPs associated with diarrhea in pig

Chr	Position	Major/Minor	P-value	Gene	Symbol
1	15179322	A/G	1.60156E-09	ENSSSCG00000004092	MTHFD1L
1	16041164	T/C	3.554E-07	ENSSSCG00000004096	-
1	16159584	A/G	1.30184E-07	ENSSSCG00000004101	LRP11
1	62436633	T/A	9.34152E-07	-	-
1	79814548	C/T	2.68493E-06	ENSSSCG00000037803	MARCKS
1	140294246	C/T	3.57494E-06	ENSSSCG00000061165	U6
1	224083486	A/C	4.91603E-06	ENSSSCG00000005257	TRPM3
1	251507385	C/T	5.08255E-06	ENSSSCG00000005455	SVEP1
1	268950815	A/G	2.21063E-06	ENSSSCG00000005654	SPTAN1
2	12900688	G/A	3.73208E-06	-	-
2	33489527	A/T	5.27261E-08	-	-
2	37046746	A/G	2.58092E-06	-	-
2	45527375	C/T	4.99807E-07	ENSSSCG00000021170	FAR1
2	120577677	G/C	1.44953E-07	ENSSSCG00000014224	SEMA6A
2	130219196	C/T	3.76914E-06	ENSSSCG00000032877	MEGF10
2	139332442	T/C	2.73601E-06	ENSSSCG00000032423	SPOCK1
2	148038240	C/T	5.21755E-06	ENSSSCG00000028976	PPP2R2B
2	150774691	T/C	4.70343E-06	ENSSSCG00000041032	-
2	150774692	G/A	4.64554E-06	ENSSSCG00000041032	-
3	2767286	A/T	3.97964E-06	ENSSSCG00000055080	-
3	6538530	G/A	1.88162E-06	ENSSSCG00000007617	ZNF655
3	6721042	C/G	1.89695E-06	ENSSSCG00000023320	-
3	10095993	G/A	4.70251E-06	ENSSSCG00000032250	SRRM3
3	12747323	A/T	5.23397E-06	-	-
3	24098874	T/G	1.01758E-06	ENSSSCG00000025266	VWA3A
3	24098883	C/T	1.02779E-06	ENSSSCG00000025266	VWA3A
3	93165828	G/A	1.31406E-06	ENSSSCG00000008428	MSH2
3	93660441	C/T	2.26473E-06	-	-
3	93746972	C/T	5.42602E-06	-	-
3	94001680	C/T	3.35637E-06	ENSSSCG00000031261	RHOQ
3	112287909	G/A	5.5036E-06	ENSSSCG00000008562	SLC35F6

3	113030154	A/C	4.3726E-06	ENSSSCG00000008575	ASXL2
3	120601175	G/A	2.56777E-06	ENSSSCG00000023910	U6
4	5237203	A/G	4.24901E-06	-	-
4	12116131	C/T	1.09208E-07	-	-
4	12412818	G/A	1.20348E-08	ENSSSCG00000046151	-
4	14177059	G/A	1.11977E-06	ENSSSCG00000052155	-
4	14208120	C/G	7.29635E-09	-	-
4	14208130	C/T	3.17866E-09	-	-
4	14261716	C/T	3.48181E-06	-	-
4	14397468	C/T	5.39177E-06	ENSSSCG00000037755	NSMCE2
4	14939020	C/T	3.65907E-06	ENSSSCG00000005975	MTSS1
4	14939024	C/T	3.44714E-06	ENSSSCG00000005975	MTSS1
4	14964386	G/T	2.04397E-06	ENSSSCG00000005975	MTSS1
4	14989799	C/T	5.0458E-07	ENSSSCG00000005975	MTSS1
4	15166150	T/A	2.19774E-06	ENSSSCG00000024412	RNF139
4	57688429	A/C	2.37432E-06	ENSSSCG00000006161	IL7
4	57790155	T/G	4.89317E-06	ENSSSCG00000006162	ZC2HC1A
4	57827801	C/A	3.16877E-06	ENSSSCG00000006162	ZC2HC1A
4	57904520	G/A	3.47909E-07	ENSSSCG00000006163	PKIA
4	58000844	C/A	5.16199E-06	-	-
4	58116223	T/C	3.5816E-06	-	-
4	58284847	T/C	8.35883E-07	-	-
4	58444552	A/T	2.15857E-06	-	-
4	58533935	C/T	7.58352E-07	-	-
4	59027912	C/T	3.57063E-06	-	-
4	59243927	C/G	1.45546E-06	ENSSSCG00000006168	PEX2
4	59243930	A/G	1.84923E-06	ENSSSCG00000006168	PEX2
4	60134704	A/G	3.40994E-06	-	-
4	60315327	T/C	5.09395E-07	-	-
4	60435875	G/A	5.43531E-06	-	-
4	60489759	A/G	6.17665E-07	ENSSSCG00000034242	HNF4G
4	60492577	C/T	7.98871E-07	ENSSSCG00000034242	HNF4G
4	60502492	G/A	1.52428E-06	ENSSSCG00000034242	HNF4G

4	60502499	G/A	2.16292E-06	ENSSSCG00000034242	HNF4G
4	60562397	T/G	1.75836E-06	ENSSSCG00000034242	HNF4G
4	60636633	G/C	2.94232E-07	ENSSSCG00000062811	-
4	60850860	C/T	3.06312E-07	ENSSSCG00000052390	-
4	60851964	T/A	5.29831E-07	-	-
4	61029259	T/C	1.44402E-06	-	-
4	61032642	G/T	3.92613E-07	-	-
4	61302628	G/A	4.76269E-06	-	-
4	61543185	T/C	1.30828E-06	-	-
4	61643850	T/C	8.56347E-07	ENSSSCG00000006174	JPH1
4	61821702	A/G	4.24573E-06	ENSSSCG00000062005	-
4	61841060	C/T	7.18486E-07	ENSSSCG00000062005	-
4	92132609	G/A	5.05903E-06	ENSSSCG00000060062	SMIM42
4	117944910	G/A	1.02069E-06	ENSSSCG00000006866	DBT
5	18863368	A/G	3.37501E-07	ENSSSCG00000062561	ATP5MC2
5	59426889	C/T	4.51799E-06	ENSSSCG00000039316	GSG1
5	64482551	A/C	3.24941E-06	-	-
5	64482593	A/G	1.71521E-06	-	-
5	65232411	A/G	3.02742E-06	-	-
5	65618796	G/A	5.26483E-06	-	-
5	65639009	G/A	2.01575E-06	ENSSSCG00000060060	KCNA6
5	65742293	A/G	1.0989E-07	ENSSSCG00000000718	GALNT8
5	65742296	A/G	1.04462E-07	ENSSSCG00000000718	GALNT8
5	65742397	T/C	1.94602E-07	ENSSSCG00000000718	GALNT8
5	77101950	C/T	3.4582E-06	ENSSSCG00000000808	SLC38A2
5	84874932	G/T	2.12566E-06	ENSSSCG00000022083	ANKS1B
5	94108274	G/A	8.25691E-07	ENSSSCG00000035495	KITLG
5	94108296	G/A	4.43339E-07	ENSSSCG00000035495	KITLG
5	101545981	C/T	5.50039E-08	ENSSSCG00000038973	PPP1R12A
6	13076165	C/G	2.17083E-07	ENSSSCG00000030420	GLG1
6	13270595	G/A	5.26363E-06	ENSSSCG00000019721	U6
6	48954120	A/G	6.23955E-07	ENSSSCG00000002998	SNRPA
6	51633005	T/C	3.32636E-06	ENSSSCG00000035706	-

6	72783231	G/A	1.3648E-07	ENSSSCG00000031836	-
6	109049927	A/G	3.07654E-08	ENSSSCG00000003712	OSBPL1A
6	130887959	T/C	1.68452E-06	-	-
6	141659054	T/C	5.89322E-07	ENSSSCG00000025085	NEGR1
6	141659065	A/G	3.87732E-07	ENSSSCG00000025085	NEGR1
6	141664256	T/C	1.13737E-06	ENSSSCG00000025085	NEGR1
6	142947281	T/A	3.78545E-06	ENSSSCG00000003793	LRRC7
6	166968467	A/T	2.78131E-06	ENSSSCG00000036909	ERI3
6	169992855	G/A	1.55436E-06	ENSSSCG00000003971	SCMH1
7	9434213	G/A	4.04236E-06	ENSSSCG00000001052	PHACTR1
7	19810059	T/C	1.10683E-06	ENSSSCG00000001097	RIPOR2
7	19861175	T/C	4.94829E-06	ENSSSCG00000001099	CMAH
7	19951669	T/C	1.36942E-06	ENSSSCG00000001099	CMAH
7	47271602	C/T	3.64344E-06	ENSSSCG00000055481	-
7	49740368	T/C	3.71488E-06	ENSSSCG00000038929	CEMIP
7	57469345	T/C	1.43325E-06	ENSSSCG00000001869	PEAK1
7	57475692	A/C	1.19151E-06	ENSSSCG00000001869	PEAK1
7	83171747	T/C	1.21926E-06	ENSSSCG00000058138	-
7	83171760	A/G	9.76086E-07	ENSSSCG00000058138	-
7	83171772	C/T	6.05345E-07	ENSSSCG00000058138	-
7	83171776	G/A	6.98614E-07	ENSSSCG00000058138	-
7	115948341	C/T	4.46052E-06	ENSSSCG00000062845	-
7	117456534	G/A	5.02001E-06	ENSSSCG00000027030	BDKRB2
7	118718588	T/G	1.75723E-09	-	-
8	5237070	C/T	2.1766E-06	ENSSSCG00000035020	STK32B
8	14661218	A/C	8.37771E-07	-	-
8	16639164	G/A	1.22078E-06	ENSSSCG00000043844	-
8	124675327	T/C	2.52517E-06	ENSSSCG00000029621	BMPR1B
8	126045536	A/G	3.66888E-06	ENSSSCG00000009197	GRID2
8	129060753	G/A	6.22497E-08	-	-
9	64000246	C/T	2.62351E-06	-	-
9	66269591	C/T	2.85588E-06	ENSSSCG00000021487	MFSD4A
9	70183337	C/T	5.16072E-06	ENSSSCG00000015301	STEAP1

9	70186714	A/G	5.26153E-07	ENSSSCG00000015301	STEAP1
9	81018115	C/G	4.3369E-07	-	-
9	81018119	C/T	5.13621E-07	-	-
9	81023767	A/C	1.356E-07	-	-
9	81947139	C/A	2.80218E-06	ENSSSCG00000022543	VWDE
9	81952879	G/C	3.27591E-06	ENSSSCG00000022543	VWDE
9	83138457	A/G	9.68355E-07	-	-
9	91088814	C/T	1.85123E-06	ENSSSCG00000015383	-
9	110030106	A/G	6.30879E-08	-	-
9	119054177	C/T	1.98343E-06	ENSSSCG00000015515	BRINP2
9	122163802	G/A	1.55694E-06	ENSSSCG00000051167	-
9	123081604	G/A	1.88442E-06	ENSSSCG00000015543	CACNA1E
9	127061905	T/G	6.21301E-08	ENSSSCG00000035595	HMCN1
9	127083221	C/T	1.10115E-07	ENSSSCG00000035595	HMCN1
9	127250512	C/T	1.27208E-07	ENSSSCG00000035595	HMCN1
9	127318476	T/C	2.58971E-06	ENSSSCG00000035595	HMCN1
9	127423890	A/G	1.48169E-07	-	-
9	127466911	A/C	7.6411E-08	ENSSSCG00000015576	TPR
9	127483264	C/A	5.46181E-08	ENSSSCG00000015576	TPR
9	127556695	T/A	1.32684E-06	ENSSSCG00000015576	TPR
9	127556701	A/G	2.25273E-07	ENSSSCG00000015576	TPR
9	127692506	A/G	4.74627E-06	-	-
9	127721925	G/T	1.35712E-06	-	-
9	127809979	T/A	4.90303E-06	-	-
9	127810040	A/C	4.10112E-07	-	-
9	127969956	A/G	4.52551E-06	ENSSSCG00000023351	PLA2G4A
9	128500275	C/A	3.47124E-06	ENSSSCG00000023451	KCNK2
9	128613696	C/T	5.27819E-08	ENSSSCG00000023451	KCNK2
9	128709259	C/A	1.85708E-06	-	-
9	128789472	C/A	3.14599E-07	-	-
9	128789473	A/C	2.90318E-07	-	-
9	137985825	C/T	4.61386E-06	-	-
9	138123269	C/T	5.47439E-07	-	-

9	138321655	G/A	2.89116E-07	-	-
9	138322628	T/C	6.31546E-09	-	-
9	138322660	C/T	3.77181E-07	-	-
9	138322680	A/C	8.42968E-07	-	-
9	138322684	C/T	9.97209E-07	-	-
9	138502726	T/A	1.00425E-06	-	-
9	138523628	T/C	1.08426E-06	-	-
9	138566617	C/T	3.63485E-06	-	-
9	138845482	T/C	1.57918E-06	-	-
9	138857892	C/G	5.40355E-06	-	-
9	138923123	C/T	9.65498E-08	ENSSSCG00000048707	-
10	25360916	G/A	1.04342E-07	ENSSSCG00000011168	ZNF510
10	25849920	C/T	4.67307E-06	ENSSSCG00000010937	HSD17B3
10	28174970	G/A	4.62607E-06	-	-
10	29929953	A/C	1.80677E-06	-	-
10	30040966	C/T	3.7631E-06	ENSSSCG00000010959	NTRK2
10	30069941	G/A	5.42098E-06	ENSSSCG00000010959	NTRK2
10	30879100	G/A	1.93901E-06	-	-
10	31013417	C/T	7.6317E-09	ENSSSCG00000021571	KIF27
10	31214759	C/T	3.7765E-06	ENSSSCG00000026087	GKAP1
10	37220582	A/G	1.36956E-07	ENSSSCG00000060194	-
10	38607839	G/T	2.38626E-06	ENSSSCG00000038811	MOB3B
11	16632891	A/G	1.16537E-06	ENSSSCG00000029598	FAM124A
11	22450262	G/C	1.32266E-07	ENSSSCG00000046928	-
11	22450263	A/G	1.85073E-07	ENSSSCG00000046928	-
11	22450288	G/A	1.58823E-06	ENSSSCG00000046928	-
11	24316171	C/T	4.75118E-06	ENSSSCG00000009428	FAM216B
11	39915388	C/T	4.07713E-06	-	-
11	64943003	A/G	1.43408E-06	ENSSSCG00000009498	-
11	67083200	C/T	4.46884E-06	ENSSSCG00000053068	U6
12	24594008	T/C	7.43538E-07	ENSSSCG00000017527	SKAP1
12	25106469	G/A	4.77592E-06	ENSSSCG00000017544	ATP5MC1
12	26822856	A/C	4.86228E-06	ENSSSCG00000027226	EPN3

12	26822874	C/T	1.41359E-06	ENSSSCG00000027226	EPN3
12	30349917	T/G	1.35771E-06	-	-
12	30971202	G/T	1.77087E-06	-	-
12	31224102	G/C	1.15981E-06	ENSSSCG00000017601	TOM1L1
12	33549633	C/T	2.15013E-06	ENSSSCG00000038505	MSI2
12	33699528	G/A	2.12974E-08	ENSSSCG00000038505	MSI2
12	41390285	C/T	4.50244E-06	-	-
12	43992153	G/A	3.10344E-06	ENSSSCG00000017753	KSR1
12	54248630	C/T	4.26028E-06	ENSSSCG00000014178	-
12	60951414	G/A	1.82144E-06	ENSSSCG00000018053	MED9
13	5796848	A/G	3.26742E-06	ENSSSCG00000049142	-
13	6098941	C/A	5.49105E-06	ENSSSCG00000011204	KCNH8
13	6521137	A/G	1.63759E-06	ENSSSCG00000011204	KCNH8
13	6843634	G/A	1.61583E-06	ENSSSCG00000011205	EFHB
13	7996328	A/C	5.51349E-07	-	-
13	8004597	G/A	3.95443E-06	-	-
13	8163365	T/A	5.02513E-06	-	-
13	8248580	A/G	1.64525E-06	ENSSSCG00000011208	ZNF385D
13	9072077	C/T	4.74523E-07	ENSSSCG00000060562	-
13	12034268	A/G	8.35081E-07	-	-
13	12034295	T/A	1.41077E-06	-	-
13	13277943	A/G	4.4188E-06	-	-
13	19254042	T/A	9.6733E-07	ENSSSCG00000011235	UBP1
13	55083993	G/A	3.71755E-06	ENSSSCG00000047664	-
13	80977105	G/A	2.17469E-06	ENSSSCG00000011666	CLSTN2
13	120559403	A/G	3.80734E-06	-	-
13	122843955	A/C	2.96361E-06	ENSSSCG00000011788	VPS8
13	129651366	A/G	3.73247E-06	ENSSSCG00000038584	FGF12
13	157547390	G/A	1.11686E-06	ENSSSCG00000021791	SENP7
13	167234389	T/C	1.89035E-06	-	-
13	175914006	T/G	1.4103E-06	ENSSSCG00000012001	ROBO1
13	186956715	T/C	3.11035E-08	-	-
13	187158847	A/C	4.86678E-06	-	-

13	189447197	T/C	4.91149E-07	ENSSSCG00000012022	APP
13	191924976	C/G	4.66324E-06	-	-
13	194833062	G/A	3.55459E-06	ENSSSCG00000012034	TIAM1
13	197213950	G/T	5.40123E-06	ENSSSCG00000028377	CRYZL1
13	205972047	C/A	2.88015E-06	ENSSSCG00000030016	PDE9A
14	5512987	T/G	2.20945E-06	-	-
14	12757143	G/C	2.23187E-07	ENSSSCG00000009682	HMBOX1
14	26354144	G/T	1.37405E-07	ENSSSCG00000009753	TMEM132C
14	55258524	T/C	1.1887E-06	ENSSSCG00000026819	NID1
14	64363473	T/G	1.11603E-06	ENSSSCG00000028327	RHOBTB1
14	64883108	C/T	3.50104E-06	ENSSSCG00000023525	TMEM26
14	120543829	A/G	1.23751E-06	ENSSSCG00000010621	ADD3
14	124791957	T/C	4.90013E-07	ENSSSCG00000010651	ABLIM1
14	126903135	T/C	5.43859E-06	ENSSSCG00000036758	HSPA12A
14	126934653	C/T	1.20256E-06	ENSSSCG00000036758	HSPA12A
14	131051006	A/G	1.17442E-07	ENSSSCG00000041462	-
15	16484629	C/T	1.12684E-06	ENSSSCG00000015694	ZRANB3
15	21874703	C/T	5.24088E-07	ENSSSCG00000015711	DPP10
15	31642937	A/G	5.13805E-06	ENSSSCG00000015741	CFAP221
15	31642956	T/C	1.41793E-06	ENSSSCG00000015741	CFAP221
15	69693306	T/C	1.97555E-06	-	-
15	89530923	T/C	3.27983E-06	-	-
15	89636588	T/C	2.24873E-06	-	-
15	135747350	C/A	3.8022E-06	ENSSSCG00000016317	AGAP1
15	135752475	G/A	1.89271E-06	ENSSSCG00000016317	AGAP1
15	136161940	C/A	5.91015E-07	ENSSSCG00000016321	IQCA1
16	397641	C/T	1.44703E-06	-	-
16	799309	C/G	1.12215E-06	ENSSSCG00000016780	CTNND2
16	1566957	C/T	1.61855E-06	-	-
16	1576321	G/A	3.17833E-06	-	-
16	1662760	C/T	1.04734E-06	-	-
16	2364009	A/G	5.14049E-07	-	-
16	2425952	G/T	9.36062E-08	-	-

16	2572019	T/A	0.000003221	-	-
16	2669207	T/C	1.48136E-06	-	-
16	2681831	A/G	9.37806E-07	-	-
16	2691805	G/A	1.80461E-06	-	-
16	2798376	A/G	4.15166E-06	-	-
16	2861949	G/A	5.35959E-07	-	-
16	2905030	C/A	5.46655E-06	-	-
16	2956386	C/T	5.28629E-06	-	-
16	2973641	A/C	1.57992E-06	-	-
16	2997122	T/A	7.25176E-07	-	-
16	2997662	A/T	3.71394E-06	-	-
16	3008513	C/T	2.66481E-07	-	-
16	3008534	G/A	3.65114E-07	-	-
16	3085488	A/G	7.72697E-07	ENSSSCG00000062918	-
16	8395840	G/T	2.70001E-06	ENSSSCG00000026842	CDH18
16	11646716	G/A	1.78256E-06	-	-
16	31108860	A/C	5.80005E-07	-	-
16	31551621	T/C	3.11361E-06	-	-
17	469798	C/T	1.7129E-06	ENSSSCG00000006966	CLDN23
17	5431615	T/A	2.88382E-06	ENSSSCG00000040013	MTUS1
17	19024157	G/A	6.41668E-07	-	-
17	21197624	G/T	1.78515E-06	-	-
17	29450451	A/G	4.86549E-06	ENSSSCG00000007112	PAX1
17	29450463	A/G	3.33641E-06	ENSSSCG00000007112	PAX1
18	797394	A/G	5.05295E-06	ENSSSCG00000026661	PTPRN2
18	3731365	C/G	2.89328E-06	ENSSSCG00000016423	DPP6
18	3877925	G/A	4.46145E-06	ENSSSCG00000016423	DPP6
18	21651145	A/C	1.9026E-06	ENSSSCG00000022865	GRM8
18	22959207	T/C	3.43026E-06	ENSSSCG00000016597	POT1
18	22959210	T/C	3.77321E-06	ENSSSCG00000016597	POT1
18	23137450	T/A	3.42823E-06	-	-
18	23216514	G/C	2.78443E-06	ENSSSCG00000041528	-
18	25060581	A/T	2.68783E-06	ENSSSCG00000016614	PTPRZ1

18	25060601	G/C	5.01372E-06	ENSSSCG00000016614	PTPRZ1
18	49154770	T/C	2.00353E-06	ENSSSCG00000016725	TNS3
X	5007008	C/T	3.85735E-06	ENSSSCG00000044694	-
X	5026779	A/G	8.44085E-08	ENSSSCG00000044694	-
X	39375169	G/A	9.83975E-07	-	-
X	41315706	T/G	3.01637E-06	ENSSSCG00000012266	SLC9A7
X	109526710	T/C	8.89473E-07	ENSSSCG00000012680	GPC3
X	122476388	G/A	4.79889E-06	ENSSSCG00000012744	CD99L2
Y	3785481	G/C	1.43326E-06	-	-
Y	3785508	T/G	2.21208E-06	-	-
Y	6485419	A/T	8.3801E-08	ENSSSCG00000051961	-
Y	6485449	G/A	8.69603E-08	ENSSSCG00000051961	-
