# Supplementary material for: Genome-wide association study and subsequent functional analysis reveal regulatory mechanism underlying piglet diarrhea
Source: Anim Biosci. 2024 Oct 28;38(4):612–28. doi: 10.5713/ab.24.0547 (PMC11917426; doi:10.5713/ab.24.0547)
Supplement: Supplementary file 2 [file ab-24-0547-Supplementary-Table-2.pdf]

Supplementary table S2.GO enrichment analysis of candidate genes(MF).

Category	Term	Count	Gene_ratio	PValue	Genes	List Tota	Pop Hits	Pop Total	Fold Enrichme	Bonferroni	Benjamini	FDR
GOTERM_MF_	GO:00055C	11	8.396946565	0.009533644	ENSSSCG0C	114	705	18939	2.592123927	0.907049325	1	1
GOTERM_MF_	GO:010252	2	1.526717557	0.011897762	ENSSSCG0C	114	2	18939	166.1315789	0.948611378	1	1
GOTERM_MF_	GO:003029	3	2.290076336	0.016515326	ENSSSCG0C	114	33	18939	15.10287081	0.983917116	1	1
GOTERM_MF_	GO:000202	4	3.053435115	0.016689742	ENSSSCG0C	114	90	18939	7.383625731	0.984609197	1	1
GOTERM_MF_	GO:000828	4	3.053435115	0.04127628	ENSSSCG0C	114	128	18939	5.191611842	0.999971161	1	1
GOTERM_MF_	GO:000804	2	1.526717557	0.063724756	ENSSSCG0C	114	11	18939	30.20574163	0.999999919	1	1
GOTERM_MF_	GO:000823	2	1.526717557	0.063724756	ENSSSCG0C	114	11	18939	30.20574163	0.999999919	1	1
