# Supplementary material for: Genome-wide association study and subsequent functional analysis reveal regulatory mechanism underlying piglet diarrhea
Source: Anim Biosci. 2024 Oct 28;38(4):612–28. doi: 10.5713/ab.24.0547 (PMC11917426; doi:10.5713/ab.24.0547)
Supplement: Supplementary file 3 [file ab-24-0547-Supplementary-Table-3.pdf]

Supplementary table S3.KEGG enrichment analysis of candidate genes

Category	Term	Descriptions	Count	Gene_Ratio	PValue	Genes	List Tota	Pop Hits	Pop Total	Fold Enrichme	Bonferroni	Benjamini	FDR
KEGG_PATHWAY	ssc04014	Ras signaling pathway	6	4.580152672	0.016068788	ENSSSCG00000015383,	59	236	9216	3.971272623	0.839669808	1	1
KEGG_PATHWAY	ssc04514	Cell adhesion molecules	4	3.053435115	0.072578804	ENSSSCG00000030420,	59	154	9216	4.057230905	0.999799413	1	1
KEGG_PATHWAY	ssc04360	Axon guidance	4	3.053435115	0.106522143	ENSSSCG00000012001,	59	182	9216	3.433041535	0.999997032	1	1
KEGG_PATHWAY	ssc04015	Rap1 signaling pathway	4	3.053435115	0.149964972	ENSSSCG00000015383,	59	213	9216	2.933396992	0.999999989	1	1
KEGG_PATHWAY	ssc04724	Glutamatergic synapse	3	2.290076336	0.160939207	ENSSSCG00000022865,	59	114	9216	4.110615522	0.999999998	1	1
KEGG_PATHWAY	ssc00591	Linoleic acid metabolism	2	1.526717557	0.208678236	ENSSSCG00000023320,	59	37	9216	8.443426477	1	1	1
KEGG_PATHWAY	ssc04072	Phospholipase D signaling path	3	2.290076336	0.241054949	ENSSSCG00000022865,	59	149	9216	3.145034695	1	1	1
KEGG_PATHWAY	ssc04530	Tight junction	3	2.290076336	0.280852239	ENSSSCG00000028976,	59	166	9216	2.822952828	1	1	1
KEGG_PATHWAY	ssc04010	MAPK signaling pathway	4	3.053435115	0.283754423	ENSSSCG00000035495,	59	295	9216	2.118012065	1	1	1
KEGG_PATHWAY	ssc04730	Long-term depression	2	1.526717557	0.311792517	ENSSSCG00000009197,	59	59	9216	5.295030164	1	1	1
KEGG_PATHWAY	ssc00140	Steroid hormone biosynthesis	2	1.526717557	0.329066651	ENSSSCG00000023320,	59	63	9216	4.958837772	1	1	1
KEGG_PATHWAY	ssc05205	Proteoglycans in cancer	3	2.290076336	0.37340803	ENSSSCG00000012680,	59	206	9216	2.274806648	1	1	1
KEGG_PATHWAY	ssc04151	PI3K-Akt signaling pathway	4	3.053435115	0.380908116	ENSSSCG00000006161,	59	351	9216	1.780095611	1	1	1
KEGG_PATHWAY	ssc03083	Polycomb repressive complex	2	1.526717557	0.405470858	ENSSSCG00000003971,	59	82	9216	3.809838776	1	1	1
KEGG_PATHWAY	ssc04024	cAMP signaling pathway	3	2.290076336	0.415877409	ENSSSCG00000038973,	59	225	9216	2.082711864	1	1	1
KEGG_PATHWAY	ssc04810	Regulation of actin cytoskelet	3	2.290076336	0.422460132	ENSSSCG00000027030,	59	228	9216	2.055307761	1	1	1
KEGG_PATHWAY	ssc05200	Pathways in cancer	5	3.816793893	0.424107561	ENSSSCG00000027030,	59	526	9216	1.484823097	1	1	1
KEGG_PATHWAY	ssc04146	Peroxisome	2	1.526717557	0.424112692	ENSSSCG00000006168,	59	87	9216	3.590882525	1	1	1
KEGG_PATHWAY	ssc04640	Hematopoietic cell lineage	2	1.526717557	0.431407505	ENSSSCG00000006161,	59	89	9216	3.510188536	1	1	1
KEGG_PATHWAY	ssc03015	mRNA surveillance pathway	2	1.526717557	0.438611488	ENSSSCG00000028976,	59	91	9216	3.433041535	1	1	1
KEGG_PATHWAY	ssc04666	Fc gamma R-mediated phagocytos	2	1.526717557	0.449249582	ENSSSCG00000037803,	59	94	9216	3.323476379	1	1	1
KEGG_PATHWAY	ssc04020	Calcium signaling pathway	3	2.290076336	0.482021156	ENSSSCG00000027030,	59	256	9216	1.830508475	1	1	1
KEGG_PATHWAY	ssc05142	Chagas disease	2	1.526717557	0.496331616	ENSSSCG00000027030,	59	108	9216	2.892655367	1	1	1
KEGG_PATHWAY	ssc04750	Inflammatory mediator regulati	2	1.526717557	0.499538991	ENSSSCG00000027030,	59	109	9216	2.866117245	1	1	1
